# JAK‐STAT pathway activation in response to spinal cord injury in regenerative and non‐regenerative stages of *Xenopus laevis*

**DOI:** 10.1002/reg2.74

**Published:** 2017-03-14

**Authors:** Victor S. Tapia, Mauricio Herrera‐Rojas, Juan Larrain

**Affiliations:** ^1^ Center for Aging and RegenerationMillennium Nucleus in Regenerative BiologyFacultad de Ciencias BiologicasPontificia Universidad Catolica de ChileSantiagoChile

**Keywords:** JAK‐STAT, Regeneration, STAT3, Spinal cord injury, *Xenopus*

## Abstract

*Xenopus laevis* tadpoles can regenerate the spinal cord after injury but this capability is lost during metamorphosis. Comparative studies between pre‐metamorphic and metamorphic *Xenopus* stages can aid towards understanding the molecular mechanisms of spinal cord regeneration. Analysis of a previous transcriptome‐wide study suggests that, in response to injury, the JAK‐STAT pathway is differentially activated in regenerative and non‐regenerative stages. We characterized the activation of the JAK‐STAT pathway and found that regenerative tadpoles have an early and transient activation. In contrast, the non‐regenerative stages have a delayed and sustained activation of the pathway. We found that STAT3 is activated in response to injury mainly in Sox2/3^+^ ependymal cells, motoneurons and sensory neurons. Finally, to study the role of temporal activation we generated a transgenic line to express a constitutively active version of STAT3. The sustained activation of the JAK‐STAT pathway in regenerative tadpoles reduced the expression of pro‐neurogenic genes normally upregulated in response to spinal cord injury, suggesting that activation of the JAK‐STAT pathway modulates the fate of neural progenitors.

## 1 INTRODUCTION

Approximately 180,000 spinal cord injury (SCI) cases are estimated to occur annually in the world (Lee, Cripps, Fitzharris, & Wing, 2014). SCI causes loss of motor and sensory functions below the lesion site, resulting in paralysis, loss of sensation, neuropathic pain and several other dysfunctions, impairing the quality of life and increasing the cost of living (Cannon, 2013; Thuret, Moon, & Gage, 2006). This type of injury produces massive cell death, including loss of neurons, astrocytes and oligodendrocytes, and almost no functional recovery is observed in mammals after SCI (Thuret et al., 2006).

Contrary to mammals, other vertebrates such as teleost fishes, urodele and anuran amphibians regenerate the spinal cord after injury (Becker, Wullimann, Becker, Bernhardt, & Schachner, 1997; Clarke, Alexander, & Holder, 1988; Filoni, Bosco, & Cioni, 1984). These model organisms are very useful to study how regenerative mechanisms such as neurogenesis and axon regeneration occur after SCI (Becker & Becker, 2015; Diaz‐Quiroz & Echeverri, 2013; Lee‐Liu, Edwards‐Faret, Tapia, & Larraín, 2013; Lee‐Liu, Méndez‐Olivos, Muñoz, & Larraín 2016; Tanaka & Ferretti, 2009). Between these regenerative models, anurans such as *Xenopus laevis* are an interesting model to study regeneration due to a stage‐dependent regenerative ability. At the beginning of metamorphosis, *X. laevis* larvae (stages 50−54) can regenerate the spinal cord while at later metamorphic and post‐metamorphic stages (stages 56−66) they lose this ability (Beattie, Bresnahan, & Lopate, 1990; Gaete et al., 2012; Gibbs, Chittur, & Szaro, 2011; Muñoz, Edwards‐Faret, Moreno, Zuñiga, Cline, & Larrain, 2015).

A comparative transcriptome‐wide analysis identified specific transcriptomic changes for regenerative (R stage) and non‐regenerative stages (NR stages) after SCI (Lee‐Liu et al., 2014). Furthermore, it was found that spinal cord regeneration proceeds through a massive and transient activation of ependymal Sox2/3^+^ cells, which have a limited response in the NR stage (Gaete et al., 2012; Muñoz et al., 2015). Although progress on the understanding of gene expression and cell response after SCI has been achieved, our knowledge on signaling pathways that could be controlling the differences in the response to SCI remains limited.

One of the signaling pathways known to control several cellular responses after SCI in mammals is the JAK‐STAT pathway. This pathway is involved in the transduction of several cytokines and growth factors (Kisseleva, Bhattacharya, Braunstein, & Schindler, 2002). Activation of the JAK‐STAT pathway leads to STAT (signal transducer and activator of transcription) phosphorylation, which causes its translocation to the nucleus to bind DNA regulatory sequences and regulate gene expression (Mertens & Darnell, 2007).

Different components of the JAK‐STAT pathway have been used to characterize pathway activation in response to SCI in mammals, such as an increase in the levels of cytokines (Pineau & Lacroix, 2007; Slaets et al., 2014; Tripathi & McTigue, 2008) or phosphorylated STAT3 (pSTAT3) (Herrmann et al., 2008; Hesp, Goldstein, Goldstein, Miranda, Kaspar, & McTigue, 2015; Tripathi & McTigue, 2008; Yamauchi et al., 2006), as well as an increase in direct targets of this pathway, such as SOCS3 (Park, Lin, & Lee, 2014). Moreover, functional studies have demonstrated that STAT3 is necessary for astrocyte response in glial scar formation, which in turn contains the inflammatory response and aids axon regeneration (Anderson et al., 2016; Herrmann et al., 2008; Okada et al., 2006; Wanner et al., 2013). In addition, gain‐of‐function studies have also shown that the JAK‐STAT pathway improves axon regeneration and collateral sprouting, enhancing motor recovery (Jin, Liu, Sun, Wang, Liu, & He, 2015; Lang, Bradley, Jacobi, Kerschensteiner, & Bareyre, 2013).

Here we aimed to characterize differences in the regulation of signaling pathways in response to SCI between the R and NR stages of *X. laevis*. We performed a pathway analysis in our previous transcriptomic database (Lee‐Liu et al., 2014) which suggested that mRNA levels of several components of the JAK‐STAT pathway are differentially regulated between the R and NR stages. Based on this, we performed a detailed characterization of the activation of the JAK‐STAT pathway in response to injury. Cytokines and *socs3* mRNA levels were measured by quantitative reverse transcription polymerase chain reaction (RT‐qPCR) and pSTAT3 levels by western blot and immunofluorescence in the spinal cord of R and NR animals after SCI. A rapid (3 hr) and transient increase (up to 2 days) in pSTAT3 was observed in Sox2/3^+^ cells, motoneurons, sensory neurons and other cell types in regenerative animals. In contrast, the analysis of NR stages showed a delayed (3−24 hr) and sustained activation of STAT3 (up to 30 days) also in Sox2/3^+^ cells. Interestingly, overexpression of a constitutively active STAT3 (caSTAT3) reduced the expression of pro‐neurogenic genes in the spinal cord after SCI, suggesting that sustained activation of the JAK‐STAT pathway could diminish neurogenesis and impair spinal cord regeneration. Our results indicate that changes in the temporal activation of the JAK‐STAT pathway are related to the loss of regenerative capacity in *X. laevis*.

## 2 RESULTS

### 2.1 Temporal activation of the JAK‐STAT pathway in regenerative and non‐regenerative stages

**Figure 1:**
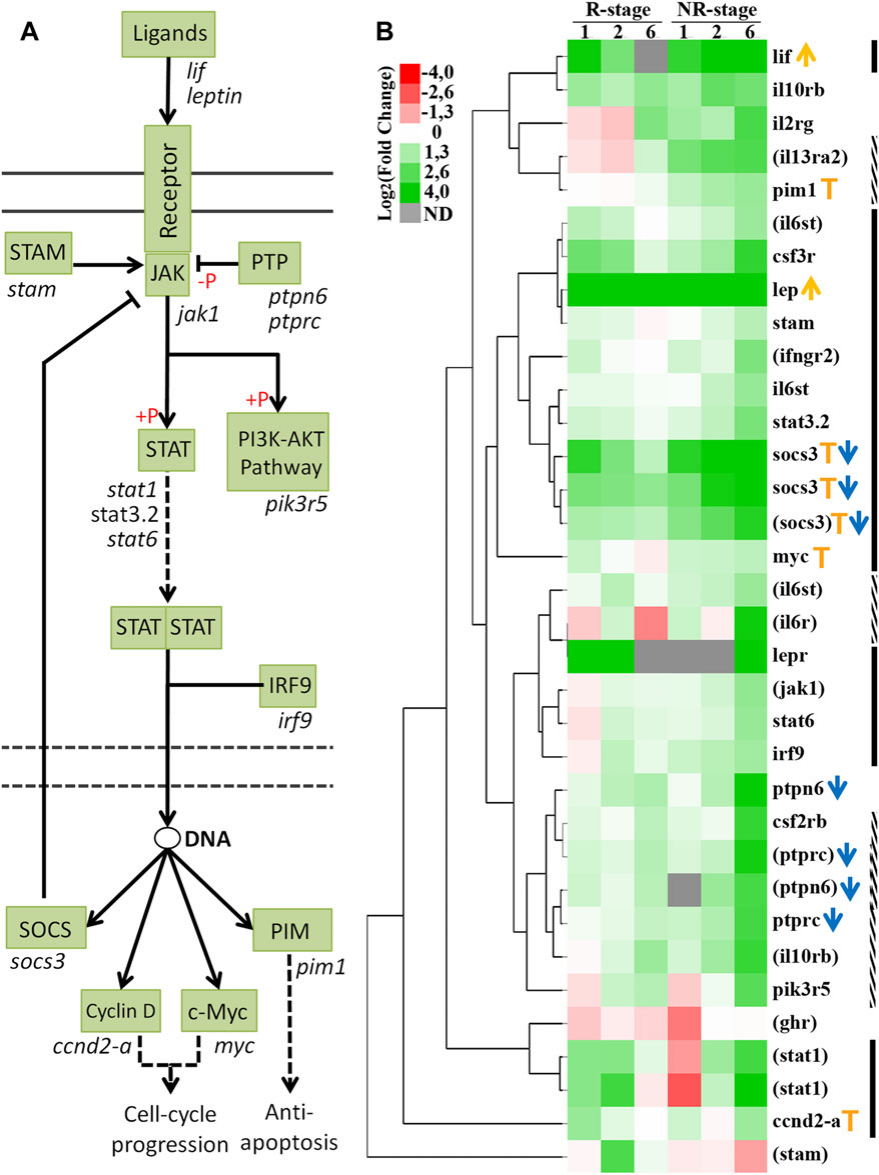
Differential regulation of components of the JAK‐STAT pathway in response to SCI in the R and NR stages. (A) Diagram of the JAK‐STAT signaling pathway based on the KEGG database. Components of the pathway are shown in green boxes and, with the exception of receptors, genes with differential expression between the R and NR stages are shown below the boxes. Following the binding of ligands to their respective receptors, STAT proteins are phosphorylated (+P red symbol) and activated by JAK tyrosine kinases. Once activated, STAT proteins dimerize and translocate to the nucleus to modulate gene expression. (B) Heat map of genes related to the JAK‐STAT pathway with differential expression between the R and NR stages. Columns indicate the expression of the R and NR stage at 1, 2, and 6 dpt; grey boxes indicate not detected (ND) transcripts. Orange arrows indicate ligands, blue arrows indicate pathway suppressors and orange T indicate pathway targets. Black lines indicate genes with an early upregulation on R stage (1 and/or 2 dpt) and a sustained or late upregulation on NR stage up to 6 dpt. Dotted lines indicate genes that are only upregulated at 6 dpt on NR stage. According to the Lee‐Liu et al. (2014) data, black and dotted lines consider only statistically significant values and genes identified by Blast2GO are shown in parentheses.

Given that our previous transcriptomic analysis shows differential regulation of biological processes between R and NR stages, we decided to perform an analysis on the same data to identify differentially regulated signaling pathways. We analyzed the transcriptome‐wide profile, which included the RNA‐Seq data from spinal cord samples from R and NR stages at 1, 2, and 6 dpt, using the KEGG pathway database (Kanehisa et al., 2004). Between the pathways observed in the KEGG analysis, we found that 36 genes related to the JAK‐STAT pathway have a differential expression between the R and NR stages (Fig. 1). From these genes, 19 were grouped in expression clusters with an early upregulation (1–2 dpt) in the R stage and a sustained or late upregulation (6 dpt) in the NR stage (Fig. 1B, black lines). Between these genes the ligands *lif* and *leptin* and targets *myc*, *ccnd‐3*, and *socs3* were included. In contrast, 10 genes were grouped in clusters with upregulation at 6 dpt on NR stages (Fig. 1B, dotted lines), including the protein tyrosine phosphatases *ptpn6* and *ptprc*. This analysis suggests that the JAK‐STAT pathway is activated differentially in the R and NR stages.

To determine which are the temporal profiles of the activation of the JAK‐STAT pathway in response to SCI in the R and NR stages, we decided to characterize the activation of the pathway by different means: (i) RT‐qPCR analysis of pathway components and (ii) western blot analysis of the phosphorylation levels of the transcription factor STAT3. Considering that some genes were already upregulated at 1 dpt in our transcriptomic analysis (Fig. 1B) we also included in our analysis earlier time points at 3 or 6 hpt. We included NR56 stage tadpoles, which is the pro‐metamorphic stage where the loss of regenerative capacity begins (Muñoz et al., 2015). Thus, the analysis of stages NR56 and NR66 led us to have a better definition of the differences between regenerative and non‐regenerative responses.

**Figure 2:**
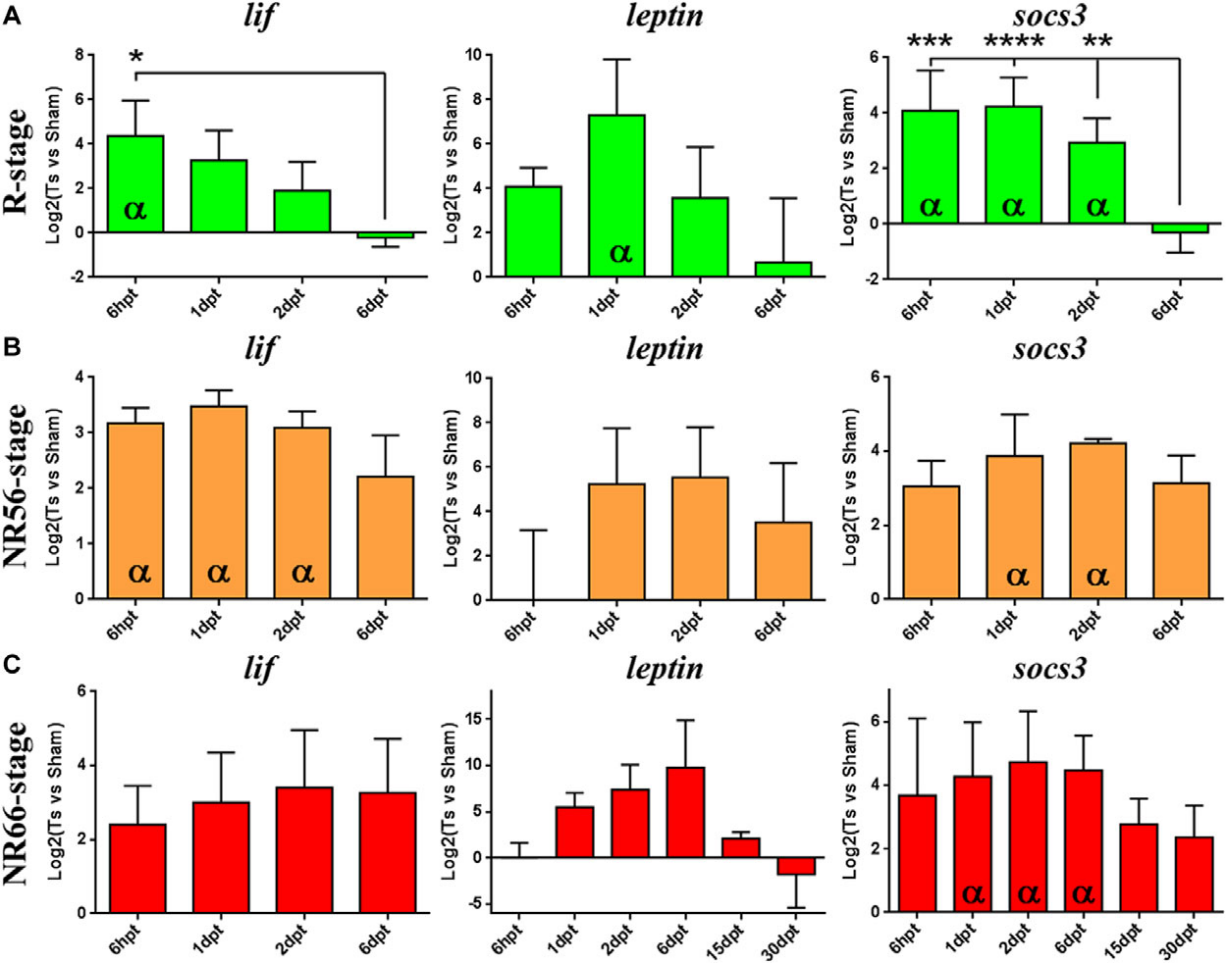
RT‐qPCR analysis of the levels of JAK‐STAT pathway components in response to SCI. Expression analyses by RT‐qPCR of genes related to the JAK‐STAT pathway are shown in response to SCI for (A) R stage (green), (B) NR56 stage (orange) and (C) NR66 stage (red). At least three biological replicates prepared from different animal pools were analyzed from 6 hpt to 6 dpt or 30 dpt for each stage. Relative expression was normalized to *eef1a‐1* expression and to sham operated animals. Two statistical tests were assessed: (i) *t*‐test with 95% confidence was performed to determine a significant difference (α symbol) from sham levels (log_2_(transected *vs* sham) = 0) and (ii) the difference between time points was assessed by multiple comparisons with a one‐way ANOVA test (****P < 0.0001; ***P < 0.001; **P < 0.01; *P < 0.05).

For RT‐qPCR analysis we measured the mRNA levels for the ligands *lif* and *leptin*, and *socs3*, a direct target of the pathway, because they appear to be regulated in the KEGG analysis (Fig. 1). In R stage animals the three mRNAs had a significant upregulation as early as 6 hpt and also at 1 dpt; this upregulation was transient, as seen by the decrease to basal levels at 6 dpt (Fig. 2A, green bars). A tendency in these mRNAs to increase at 6 hpt and 1 dpt in response to injury in NR stages was also observed, although only statistically significant increases were observed for *lif* in the NR56 stage and for *socs3* in both NR stages, but contrary to the regenerative tadpoles no return to basal levels was observed at 6 dpt (Fig. 2B, C, orange and red bars). To determine the extent of this upregulation at the NR66 stage, the levels of the three mRNAs were also analyzed at 15 and 30 dpt. mRNA for *lif* was not detected in sham and transected animals at 15−30 dpt. Downregulation of *leptin* levels was observed and, although *socs3* transcripts showed a tendency to decrease between 15 and 30 dpt, no definitive return to basal levels was observed (Fig. 2C).

**Figure 3:**
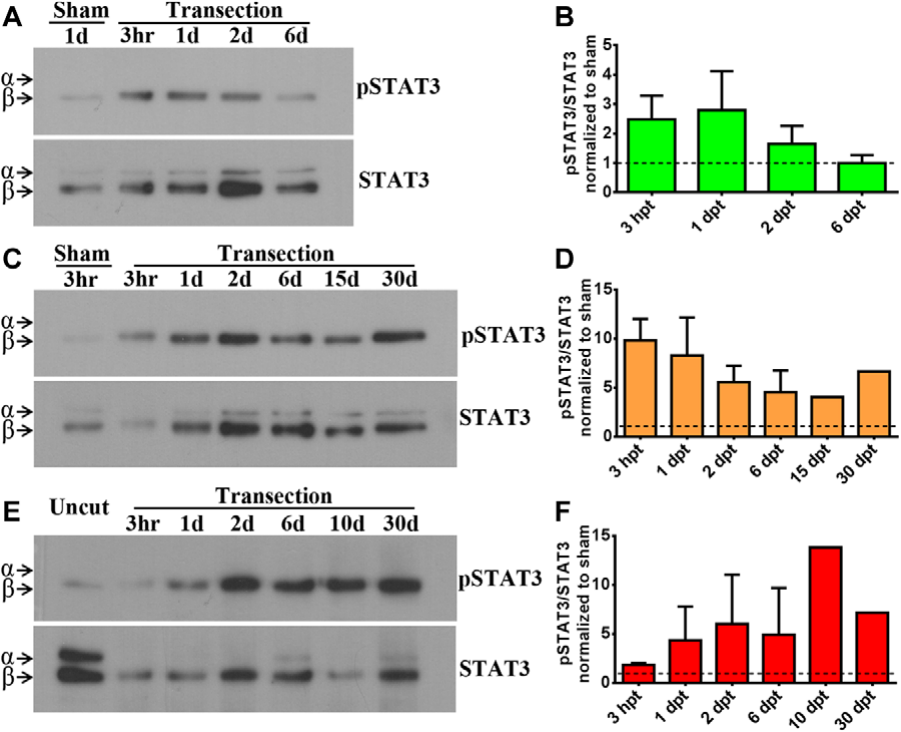
Western blot analysis of the activation of the JAK‐STAT pathway in response to SCI. Western blot and histogram analyses showing the levels of phosphorylated STAT3 (pSTAT3) and total STAT3 in the spinal cord after injury and compared with a sham or uncut controls for (A), (B) R stage, (C), (D) NR56 stage and (E), (F) NR66 stage. The presence of STAT3α and STAT3β isoforms is indicated by arrowheads. Western blots are representative of three independent assays for R stage and two assays for each NR stage through 3 hpt and 6 dpt; later times are unique samples. Histograms show the pSTAT3/STAT3β ratio normalized to controls. Control levels (ratio = 1) are indicated by dashed lines. No difference between time points was observed by multiple comparisons with a one‐way ANOVA test.

In addition to the RT‐qPCR results, western blot analyses against pSTAT3 and total STAT3 were performed to evaluate the activation of the pathway (Fig. 3). STAT3 analysis showed two isoforms recognized as STAT3α and STAT3β, the latter being 55 amino acids smaller. Although both isoforms can be phosphorylated and translocated to the nucleus to induce gene regulation (Maritano et al, 2004; Huang et al, 2007), in our experiments we only detected phosphorylation of the STAT3β isoform. In the R stage SCI induced STAT3 phosphorylation as early as 3 hpt and STAT3 remained phosphorylated until 2 dpt, decreasing to basal levels at 6 dpt (Fig. 3A, B). In contrast, in the NR stages increase of pSTAT3 levels was first detected between 3 hpt and 1 dpt and remained phosphorylated at least until 30 dpt (Fig. 3C−F).

In summary, we have found a transient activation of the JAK‐STAT pathway in response to SCI in R stage animals and a slightly delayed and sustained activation in NR stages, suggesting a possible correlation between the duration of JAK‐STAT activation and regenerative capacities.

### 2.2 Tissue‐specific activation of the JAK‐STAT pathway

**Figure 4:**
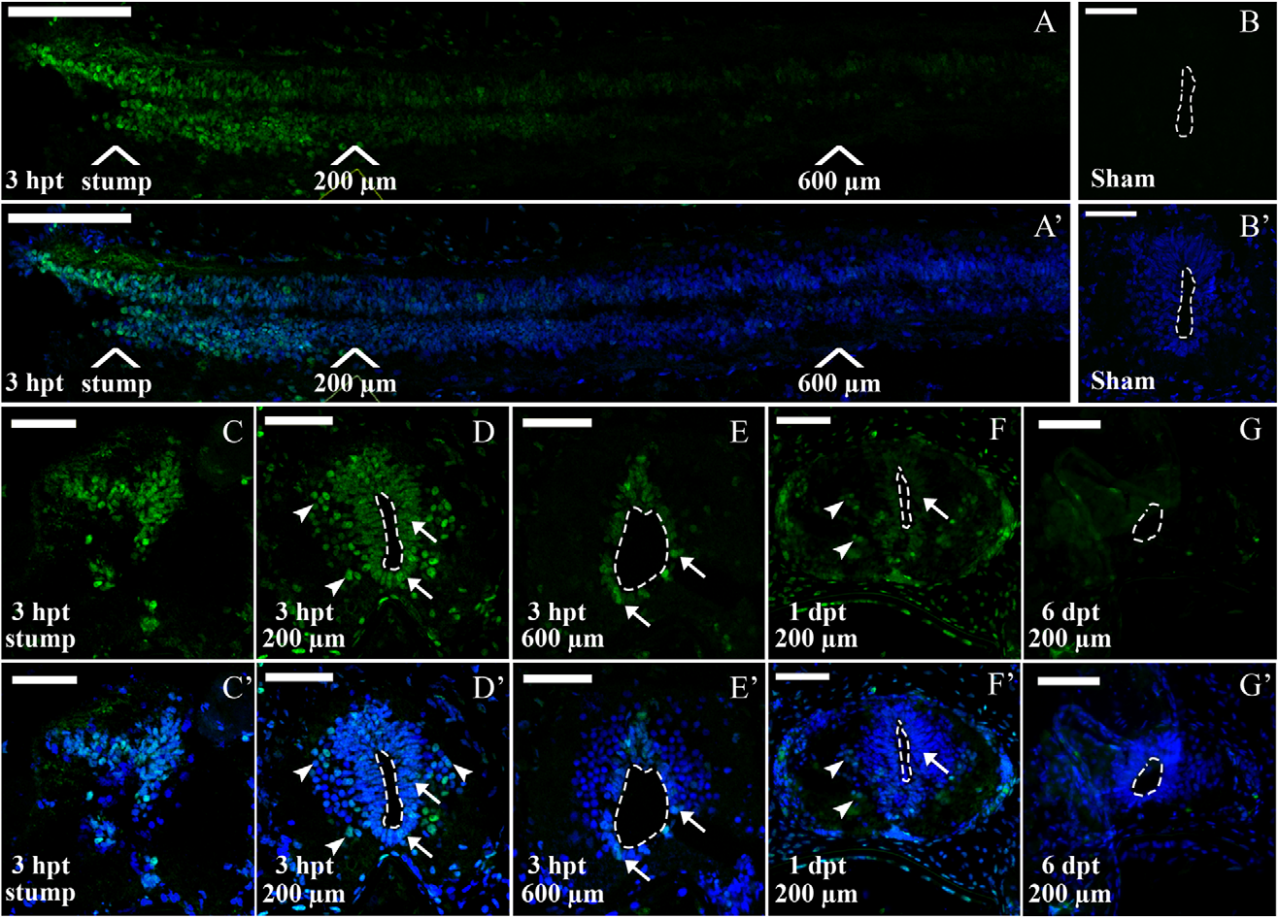
Immunofluorescence analysis of p‐STAT3 in R stage animals after SCI. Sections through the spinal cord of regenerative animals analyzed for pSTAT3 (green) and TOTO3 (nuclei, blue). (A), (A′) Sagittal section through the caudal portion of a spinal cord at 3 hpt. White arrows show positions for the stump and 200−600 μm caudal to the lesion site. (B), (B′) Coronal section from an uninjured spinal cord. (C)−(E′) Coronal sections from a transected animal at 3 hpt at (C), (C′) the stump level and (D), (D′) 200 and (E), (E′) 600 μm caudal to the lesion site. Arrows indicate representative pSTAT3^+^ cells in the ventricular zone (white arrows) and in the grey matter (white arrowheads). (F), (F′) Coronal sections at 1 dpt at 200 μm caudal to the injury site. (G), (G′) Coronal section at 6 dpt at 200 μm caudal to the injury site. Dotted lines delimit the central canal. White bars indicate 100 μm (A), (A′) and 50 μm (B)−(G′). The sections depicted are representative of three animals per time point.

Considering the temporal differences in the activation of the JAK‐STAT pathway between the R and NR stages, we decided to determine in which tissues this pathway is activated in response to injury. For this we performed immunofluorescence detection of pSTAT3 in spinal cord cryosections. Sagittal sections of R stage animals at 3 hpt showed a strong increase of nuclear pSTAT3 signal compared to sham operated animals (Fig. 4A−B′). Detailed analysis of pSTAT3 in coronal sections at different distances from the injury site demonstrated a gradient of activation: at 3 hpt the pSTAT3 signal has a massive presence in the stump of the spinal cord (Fig. 4C, C′), at 200 μm it is mainly activated in the ventricular zone (VZ) and in some scattered cells in the grey and white matter (Fig. 4D, D′), and at 600 μm it is only present in the ventricular layer at lower levels (Fig. 4E, E′). Immunofluorescence in coronal sections at 200 μm caudal to the spinal cord stump supported the western blot analysis, showing that STAT3 is still phosphorylated at 1 dpt and no signal was observed at 6 dpt (Fig. 4F−G′).

**Figure 5:**
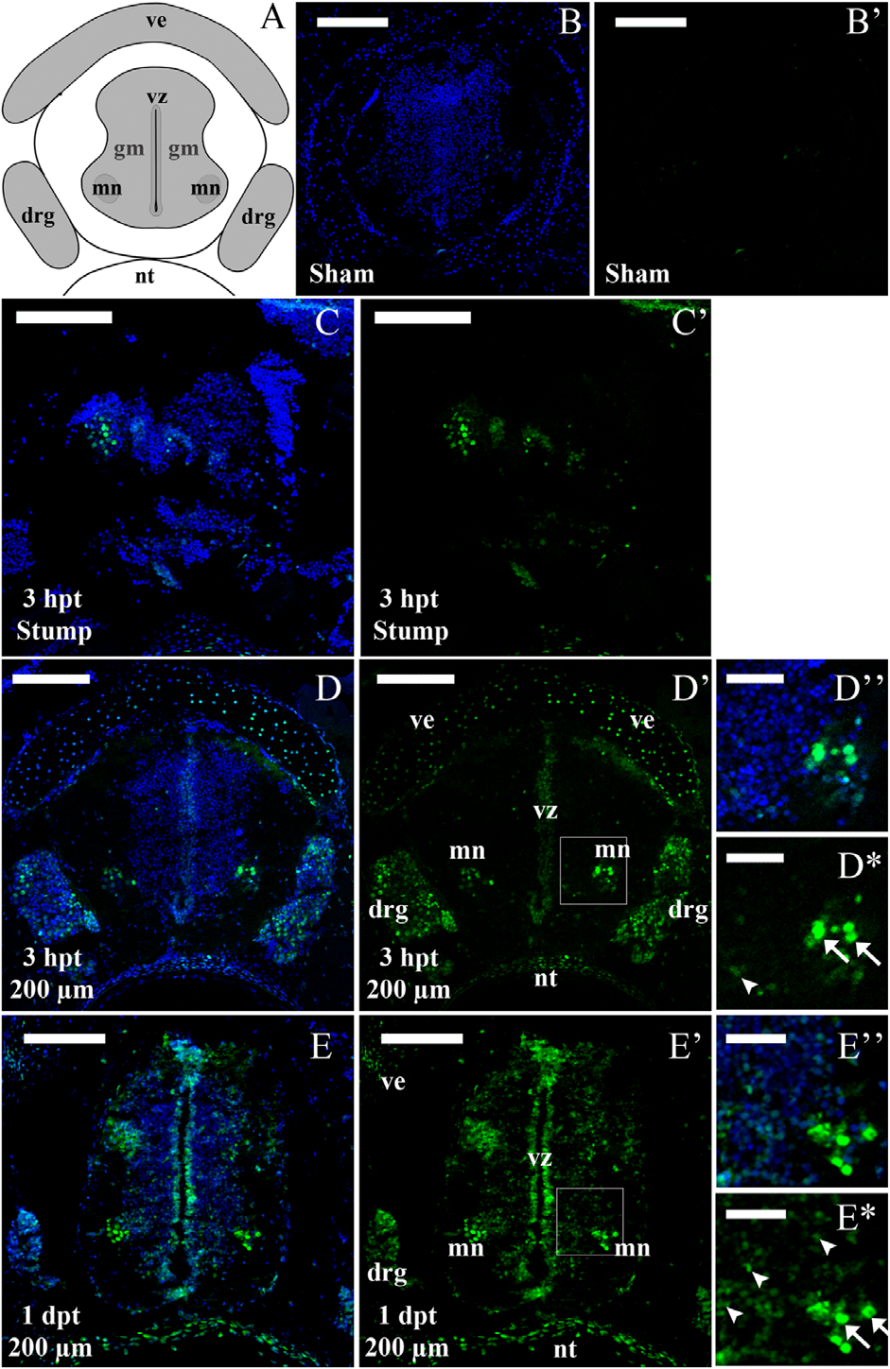
Immunofluorescence analysis of pSTAT3 levels in NR56 stage animals after SCI. (A) Scheme representing a coronal section of the spinal cord from an NR56 animal: Vz, ventricular zone; Mn, motor neuron columns; Gm, grey matter; Ve, vertebra; Drg, dorsal root ganglion; Nt, notochord. (B)−(E*) Coronal sections through the spinal cord, analyzed for immunostaining of pSTAT3 (green) and TOTO3 staining (nuclei, blue). (B), (B′) Section from a sham operated animal at 1 dpt. (C) (C′) Section from the spinal cord stump at 3 hpt. (D)−(E*) Sections at 3 hpt (D), (D*) and 1 dpt (E), (E*) 200 μm caudal to the lesion; boxed areas indicate a digital zoom of grey matter and motoneuron columns. Arrows indicate representative pSTAT3^+^ motoneurons (white arrows, cells with large nuclei) and cells in the grey matter (white arrowheads). White bars indicate 100 μm (B)−(D′), (E), (E′) and 30 μm (D″), (D*), (E″), (E*). The sections depicted are representative of two animals per time point.

Immunofluorescence analysis for pSTAT3 was also performed in coronal sections of the spinal cords from NR56 stage animals (Fig. 5A′). In control animals 1 day after sham operation no signal was observed (Fig. 5B, B′). At 3 hpt pSTAT3^+^ cells were observed in the stump (Fig. 5C, C′) although not as many as for R stage animals (Fig. 4C, C′); at 3 hpt at 200 μm caudal to the injury site, cells in the VZ showed low levels of pSTAT3 and a strong signal was detected in motoneuron columns and dorsal root ganglion (DRG) (Fig. 5D, D*). Interestingly, compared to the response at 3 hpt, higher levels of pSTAT3 were observed in cells of the VZ and the grey matter at 1 dpt and the JAK‐STAT pathway remained active in the motoneurons and DRG (Fig. 5E, E*). At 3 hpt and 1 dpt pSTAT3^+^ cells were also found outside the spinal cord in the vertebrae and tissue limiting the notochord (Fig. 5D, D′, E, E′).

**Figure 6:**
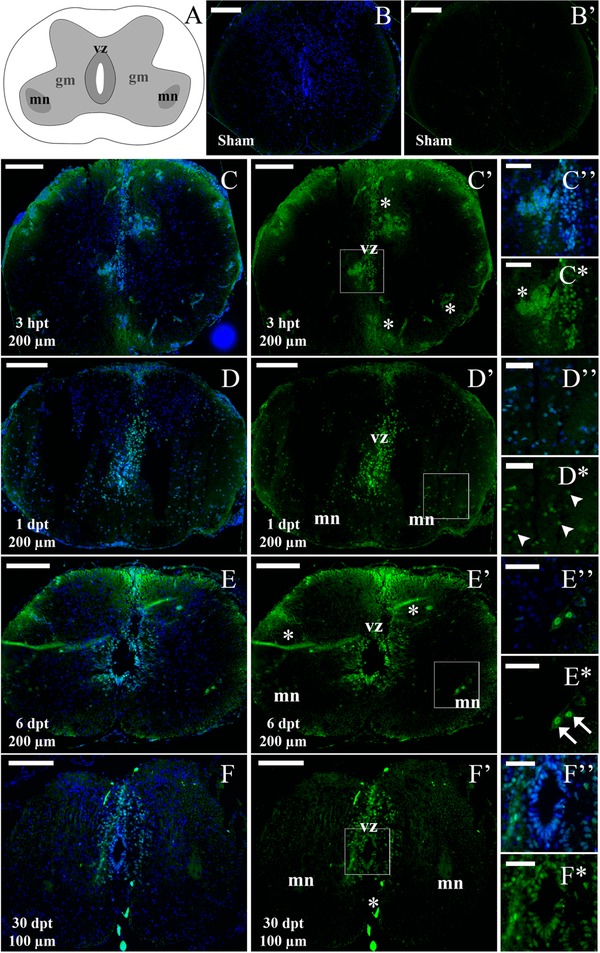
Immunofluorescence analysis of pSTAT3 levels in NR66 stage animals after SCI. (A) Scheme representing a coronal section from an NR66 stage spinal cord: Vz, ventricular zone; Mn, motor neuron columns; Gm, remaining grey matter. (B)−(F*) Coronal sections through the spinal cord, analyzed for immunostaining for pSTAT3 (green) and TOTO3 staining (nuclei, blue). (B), (B′) Section from a sham operated animal at 1 dpt. (C), (C*) Section from a transected animal at 3 hpt and 200 μm caudal to the lesion; boxed area indicates a digital zoom of the ventricular zone (vz) and auto‐fluorescent clot (asterisk). (D), (E*) Section at 1 dpt (D), (D*) and 6 dpt (E), (E*) 200 μm caudal to the lesion; boxed areas indicate digital zooms of grey matter and motoneuron columns. Arrows indicate representative pSTAT3^+^ motoneurons (white arrows, cells with large nuclei) and cells in the grey matter (white arrowheads). (F), (F*) Section at 30 dpt and 100 μm caudal to the lesion; boxed area indicates a digital zoom of the ventricular zone. White bars indicate 100 μm (B), (C′), (D), (D′), (E), (E′), (F), (F′) and 30 μm (C″), (C*), (D″), (D*), (E″), (E*), (F″), (F*). The sections depicted are representative of three animals for 3 hpt to 6 dpt and two animals for 30 hpt.

For the analysis of NR66 stage animals the spinal cords were separated from the vertebrae before processing; therefore external tissues such as the DRG and vertebrae were not analyzed (Fig. 6A). No signal for pSTAT3 was detected on sham operated animals (Fig. 6B, B′), but at 3 hpt pSTAT3^+^ cells were present in the VZ (Fig. 6C, C*, Fig. S2). Auto‐fluorescent signal from clots and blood vessels was observed on sections from NR66 stage animals because of increased bleeding after the injury. This signal is not specific because it was also detected in sections stained without the primary antibody (Fig. S2) and it is not localized on the nucleus (Fig. 6Cᶧ, C*). At 1 and 6 dpt more pSTAT3^+^ cells were detected in the VZ, the grey matter and the motoneuron columns (Fig. 6D−E*). In line with the western blot analysis at 30 dpt pSTAT3^+^ cells were still present in regions close to the lesion site (50−150 μm) in the VZ (Fig. 6F, F*).

In summary, we have found that at early times there is a massive presence of pSTAT3^+^ cells in R stage stumps. Activation in R stage animals is higher in the regions close to the injury site and a gradient of activation is observed up to a distance of 600 μm from the injury mainly on the VZ and in some nucleus on the grey matter. In NR stages pSTAT3 is found in the VZ, motoneuron columns and DRG. As demonstrated by RT‐qPCR and western blot analysis, pSTAT3 staining gives further support for the differences in the temporal activation of the JAK‐STAT pathway, which shows that it is very rapid and transient in the R stage but sustained for longer times in NR stages.

### 2.3 The JAK‐STAT pathway is activated in Sox2/3^+^ ependymal cells and neurons

**Figure 7:**
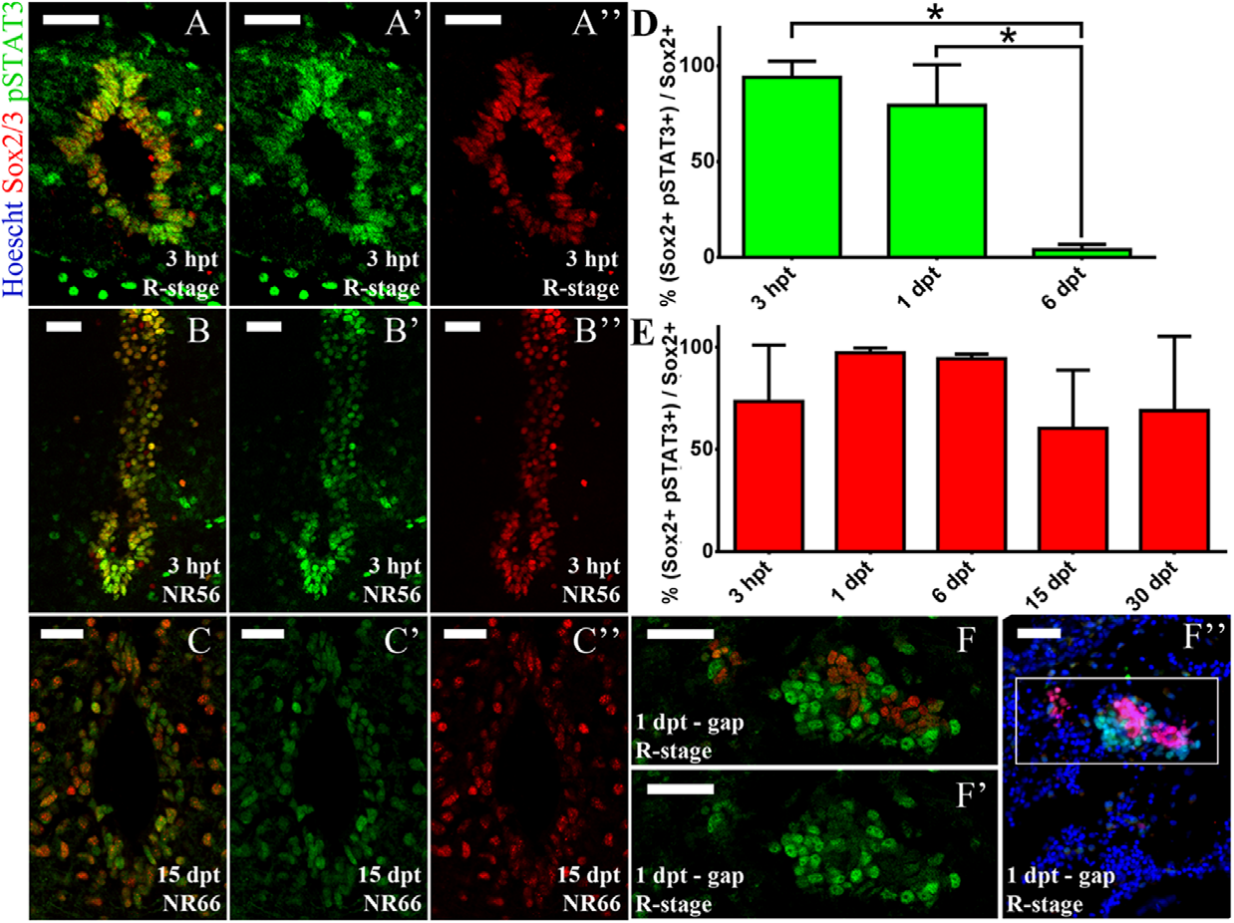
JAK‐STAT pathway activation in Sox2/3^+^ cells. Coronal sections through the spinal cord and injury site of R, NR56 and NR66 animals, analyzed for pSTAT3 (green), Sox2 (red) and Hoechst (nuclei, blue). (A)−(B″) Sections from R stage (A)−(A″) and NR56 stage (B)−(B″) animals at 3 hpt showing the ventricular zone at 300 μm caudal to the lesion site. (C)−(C″) Section from an NR66 stage animal at 15 dpt showing the ventricular zone at 100 μm caudal to the lesion site. The sections depicted are representative of two to three animals. (D), (E) Quantification of ventricular pSTAT3^+^ Sox2^+^ cells in one section per animal was performed for (D) R and (E) NR66 stages. Difference between time points was assessed by multiple comparisons with a one‐way ANOVA test (*P < 0.1). (F)−(F’’) Section from an R stage animal at 1 dpt showing the lesion site (ablation gap); representative of three animals. (F), (F′) An optical zoom of (F″). White bars indicate 30 μm.

In order to determine in which cells the JAK‐STAT pathway is activated in response to SCI we performed double immunofluorescence against pSTAT3 and cell‐type specific markers. We have shown previously that, in regenerative tadpoles, Sox2/3^+^ ependymal cells from the VZ proliferate after SCI and are necessary for spinal cord regeneration (Gaete et al., 2012; Muñoz et al., 2015). Considering that we detected pSTAT3^+^ cells in the VZ of the three stages analyzed, we decided to evaluate whether the JAK‐STAT pathway is activated in Sox2/3^+^ cells. We found that pSTAT3 co‐localized with Sox2/3^+^ cells in the R and NR stages (Fig. 7A−C″). Quantification of pSTAT3 and Sox2/3 co‐localization in the VZ showed an early and transient increase of pSTAT3^+^ Sox2/3^+^ cells in the R stage, but a sustained activation in the NR66 stage up to 1 month (Fig. 7D, E). Previously, we also showed that Sox2/3^+^ cells migrate to the ablation gap in the R stage (Muñoz et al., 2015); here we found that the Sox2/3^+^ cells present in the ablation gap were negative for pSTAT3 and were surrounded by pSTAT3^+^ cells (Fig. 7F−D″).

Considering that we found a pSTAT3 signal in the grey matter, motoneuron columns and DRG, we also analyzed JAK‐STAT activation in motoneurons and sensory neurons using an anti‐Islet1/2 antibody, a marker for both motoneurons in the spinal cord and sensory neurons in the DRG (Uemura et al., 2005). We found transient levels of pSTAT3 in Islet1/2^+^ motoneurons and sensory neurons in R stage animals at 3 hpt, which were then reduced at 1 dpt (Fig. S3A−B″). In contrast, in NR56 stage animals Islet1/2 was not expressed in motoneurons but was expressed at 3 hpt and 1 dpt in DRG and co‐localized with pSTAT3 (Fig. S3C−D″).

Lastly, as JAK‐STAT also controls neuro‐inflammation (Molet et al., 2015; Qin et al., 2012) we decided to characterize the levels of pSTAT3 in inflammatory cells using an antibody against CD45, a marker for leukocytes (Barritt & Turpen, 1995) that allows the identification of microglia and infiltrated cells. In R stage animals we found STAT3 activation on CD45^+^ cells, mainly located in the meninges (Fig. S4A, B′). This activation was transient but not rapid, and went up to 54% of pSTAT3^+^ CD45^+^ leukocytes at 1 dpt (Fig. S4C). In the NR66 stage a transient activation was also observed at 1dpt, but few positive cells were observed in the meninges and only 19% of the leukocytes were pSTAT3 positive (Fig. S4D−F).

In summary we found stage‐dependent differences in JAK‐STAT activation in relevant cell types for spinal cord regeneration. The regenerative stage has an early and transient activation in Sox2/Sox3^+^ ependymal cells, Islet1/2^+^ motoneurons and sensory neurons in response to SCI. Besides, the results suggested that the JAK‐STAT pathway also has a dynamic spatial control, as Sox2/Sox3^+^ cells populating the injury gap are negative for pSTAT3. In NR stages pSTAT3 also co‐localizes with Sox2/3 ependymal cells and Islet1/2 in the DRG. Interestingly the JAK‐STAT pathway is activated in meningeal leukocytes but few cells are observed in white and grey matter of the R and NR stages.

### 2.4 The JAK‐STAT pathway regulates the levels of expression of neurogenic genes during spinal cord regeneration

Our results suggested a strong correlation between changes in the spatio‐temporal activation of JAK‐STAT and the regenerative ability of *X. laevis*. To test the impact of the sustained activation of the JAK‐STAT pathway during spinal cord regeneration we prepared the transgenic line *Tg(HS:caSTAT3GR)* that allowed the regulated expression of a constitutively activated version of STAT3 (caSTAT3) (Fig. S5A), which was previously used in *X. laevis* to study STAT3 function in early development (Nichane et al., 2010). The temporal expression of the caSTAT3 construct can be tightly regulated by two mechanisms: (i) transcriptional control by the heat shock promoter and (ii) its nuclear translocation because it is fused to a glucocorticoid domain that allows nuclear translocation in response to the presence of dexamethasone.

For experiments an F0 founder carrying the transgene *HS:caSTAT3GR* was outcrossed to WT animals. The F1 offspring had a WT‐to‐transgenic ratio of 1:7 tadpoles, indicating that several copies of the construct were inserted in the F0 founder. Animals were raised to R stage, heat shocked and incubated with dexamethasone. Transgene expression was verified by RT‐qPCR and by western blot against STAT3‐GR (Fig. S5B, C). Activation of the JAK‐STAT pathway was verified by RT‐qPCR, where we observed an increase in *socs3* of 14 times over WT animals (Fig. S5D). Activation of caSTAT3 for only 2 days resulted in a rapid loss of half of the animals making functional experiments in whole animals infeasible (Fig. S5E). Similar results were obtained when an F1 transgenic animal was outcrossed to WT animals. The F2 offspring had a WT‐to‐transgenic ratio of 1:2, suggesting that by Mendelian inheritance the offspring had only one or two copies of the transgene. Nevertheless, upon caSTAT3 activation the animals also had a low survival rate indicating that the copy number of the transgene was not the cause of mortality.

**Figure 8:**
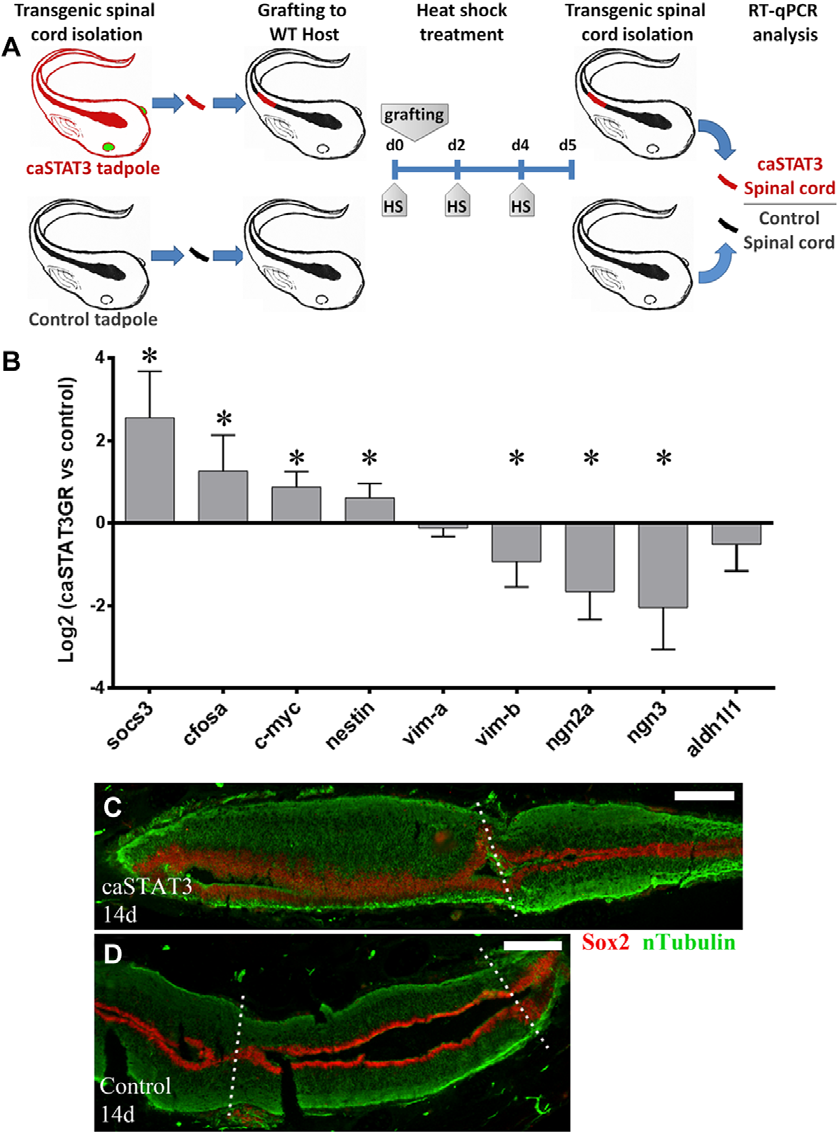
Sustained JAK‐STAT pathway activation reduces neurogenesis. (A) Diagram of the spinal cord graft approach is shown. The caudal portion of the thoracic spinal cord was isolated from transgenic tadpoles (identified by GFP^+^ eyes and represented as red tadpoles) or their control siblings (black tadpoles), and then it was grafted into WT hosts (black). First heat shock was done 30 min before the spinal cord grafting, and then every 2 days. After 5 days the spinal cord grafts were isolated and analyzed by RT‐qPCR. (B) RT‐qPCR analysis for STAT3 targets (*socs3*, *c‐fosa*, and *c‐myc*), NSPC markers (*nestin, vima*, and *vimb*), neurogenic genes (*ngn2a* and *ngn3*) and astrocyte marker (*aldh1l1*). Five biological replicates prepared from different animal pools were analyzed. One sample *t*‐test with 95% confidence was performed to determine a significant difference (* symbol) from control grafts. (C), (D) Representative animals treated as indicated in (A) were analyzed at 14 days post‐grafting. Longitudinal sections of transgenic (C) and control (D) grafts were analyzed by immunofluorescence. Central canal continuity was analyzed by Sox2/Sox3 (red) and axon continuity by nTubulin (green). In (C) transgenic graft (left side of the figure) is connected (indicated by a dotted line) to the caudal portion of the host spinal cord (right side). The section plane of (C) do not include the connection to the rostral side of the host. In (D) control graft is observed in the middle and connected (dotted lines) to the rostral and caudal portions (left and right sides) of the host spinal cord. White bars indicate 30 μm. The sections depicted are representative of three control and transgenic grafts.

To overcome this problem, we developed spinal cord transplantation experiments to achieve localized caSTAT3 expression only in the spinal cord. For this a piece of the spinal cord of 1.5–2.0 μm was isolated from F2 *Tg(HS:caSTAT3GR)* tadpoles at R stage and transplanted into WT animals (Figs. 8A, S1D). For control similar transplantation experiments were done using the WT siblings of *Tg(HS:caSTAT3GR)* as donors. The animals received a first heat shock before the transplant and then a heat shock at days 2 and 4, were incubated with dexamethasone for 5 consecutive days, and then transplanted spinal cords were isolated for RT‐qPCR analysis (Fig. 8A). Using this approach we were able to induce sustained JAK‐STAT pathway activation in the *Tg(HS:caSTAT3GR)* spinal cord as verified by the significant increase of the mRNA levels of *socs3* but also of *c‐fosa* and *c‐myc* (Fig. 8B), two other STAT3 direct targets (Bowman et al., 2001; Dauer et al., 2005; Lo et al., 2005; Yang, Lerner, Besser, & Darnell, 2003). Transplanted animals survived the treatments and the spinal cord grafts established rostrocaudal continuity with the host spinal cord (Fig. 8C); therefore this approach allowed us to induce a sustained induction of the JAK‐STAT pathway in a regenerative process similar to the previously characterized SCI providing an experimental setup to analyze the function of the JAK‐STAT pathway.

It has been demonstrated that the JAK‐STAT pathway modulates the maintenance of neural and progenitor stem cells (NSPC) (Hong & Song, 2015; Yoshimatsu et al., 2006) and gliogenic differentiation (Barnabé‐Heider et al., 2005; He et al., 2005) during nervous system development. Based on the finding that the JAK‐STAT pathway is activated in Sox2/3^+^ ependymal cells, on the facts that in R stage SCI activates the expression of the neurogenic genes *neurogenin2*, *neurogenin3*, and *NeuroD*, and that neurogenesis is probably necessary for spinal cord regeneration in R stage animals (Muñoz et al., 2015), we decided to test the effects of a sustained activation of this pathway on the expression of NSPC, glial and neurogenic markers. Regarding NSPC we evaluated the levels of the intermediate filaments Nestin and Vimentin and found that sustained activation of the JAK‐STAT pathway resulted in a significant increase in *nestin* mRNA, no change in *vimentinA* and a decrease in *vimentinB* (Fig. 8B).

Importantly, the mRNA levels for the neurogenic genes *Neurogenin2a* and *3* (Bertrand, Castro, & Guillemot, 2002) had a reduced expression when the activation in the spinal cord of the JAK‐STAT pathway was sustained (Fig. 8B). This seemed to be specific for neurogenic genes because the glial marker Aldh1l1 did not have a significant regulation (Fig. 8B). These results suggest that a tight regulation of the activation of the JAK‐STAT pathway is required for proper neurogenesis during spinal cord regeneration in R stage *X. laevis*.

## 3 DISCUSSION

In this study, we performed bioinformatics analysis of previous transcriptomic data to identify signaling pathways that were differentially regulated in response to SCI in the R and NR stages in *X. laevis*. The differential activation of the JAK‐STAT pathway was identified and followed by a detailed characterization of its activation and possible function. We found that in the R stage there is a rapid activation of the JAK‐STAT pathway at 3 hr post injury, mainly in Sox2/Sox3^+^ ependymal cells and neurons. This activation is transient; at 1−2 dpt it is already reduced and at 6 days after injury it returns to its basal levels. In NR stages the activation is also localized to Sox2/3^+^ cells and neurons, but the temporal profile is different to R stages. Its activation is slightly slower, occurs between 3 and 24 hr post injury, but more importantly the activation is sustained and lasts at least for 30 days. Sustained activation of the JAK‐STAT pathway in spinal cords overexpressing a caSTAT3 construct reduced the expression of neurogenic genes suggesting that neurogenesis can be disrupted when this pathway is maintained active for long periods of time.

### 3.1 JAK‐STAT pathway and early regenerative response

The early and transient activation of the JAK‐STAT pathway in regenerative tadpoles suggests a role of STAT3 in the early regenerative response. In mammals, STAT3 controls the maintenance of NSPCs in development and adult neurogenesis (Hong & Song, 2015; Müller, Chakrapani, Schwegler, Hofmann, & Kirsch, 2009; Yoshimatsu et al., 2006), and in a context of neural regeneration JAK‐STAT cytokines induce Müller glia proliferation and reprogramming in zebrafish retina (Zhao, Wan, Powell, Ramachandran, Myers, & Goldman, 2014). Here we show that STAT3 activation on Sox2/Sox3^+^ cells precedes transcriptome changes associated with the cell cycle (Lee‐Liu et al., 2014) and the proliferative response of Sox2/Sox3^+^ cells (Muñoz et al., 2015). In addition, we have shown that caSTAT3 induces the expression of *c‐myc*, which controls NSPC proliferation and differentiation (Kerosuo et al., 2008; Zinin et al., 2014), and *nestin*, which is necessary for NSPC maintenance (Park et al., 2010). Therefore, our results suggest that STAT3 is an early activator of Sox2/Sox3^+^ cell response in SCI, which is a necessary factor for R stage spinal cord regeneration (Muñoz et al., 2015).

Another possible mechanism regulated by the JAK‐STAT pathway could be reconnection of the severed spinal cord. Our previous study showed that, in R stage animals, ependymal‐like cells invade the ablation gap and then rostrocaudal continuity of the spinal cord is reestablished (Muñoz et al., 2015). This behavior is unique for regenerative models, as similar responses are observed in zebrafish and newts (Goldshmit, Sztal, jusuf, Hall, Nguyen‐Chi, & Currie, 2012; Muñoz et al., 2015; Zukor, Kent, & Odelberg, 2011) but not in *X. laevis* NR stages (Muñoz et al., 2015). We show that a massive increase of pSTAT3 levels occurs through the stump of regenerative tadpoles. In contrast, fewer pSTAT3^+^ cells are observed in the spinal cord stumps of NR stages. In addition, an intricate spatial pattern of activation is present in the ablation gap of the R stage, where pSTAT3^+^ cells surround Sox2/3^+^ cell clusters. The unique activation pattern of the R stage in the spinal cord stump and ablation gap suggests that early activation of the JAK‐STAT pathway controls the reestablishment of spinal cord continuity.

### 3.2 Activation of the JAK‐STAT pathway in neurons

Another possible mechanism for functional recovery after SCI that could be related to the JAK‐STAT pathway is axon regeneration. The regenerative stages of *X. laevis* are able to regenerate axons in response to SCI but this capacity is lost during metamorphosis (Gibbs et al., 2011). Conversely, STAT3 is a necessary factor for successful axon regeneration (Bareyre, Garzorz, Lang, Misgeld, Büning, & Kerschensteiner, 2011). We evaluated differences in the neuronal pSTAT3 levels to determine a relation between activation of the JAK‐STAT pathway and axon regeneration.

We used markers Islet1/2 to analyze motoneurons and sensory neurons, but we could not compare R and NR stages because Islet1/2^+^ motoneurons decrease in number and intensity signal during metamorphosis. In mice and chicken, Islet1 is highly expressed on motoneuron differentiation during spinal cord development, but then the expression decreases to a reduced mature motoneuron number (Kim, Park, Jeong, & Song, 2015; Kobayashi, Nakano, Atobe, Kadota, & Funakoshi, 2011). Therefore, we hypothesize that Islet1/2 marker led us to identify an immature pool of motoneurons in the R stage. In contrast, in the NR stages due to the scarce Islet1/2^+^ signal we recognized motoneurons by anatomical properties corresponding to motoneuron columns.

Here we show that both the R and NR56 stage have an early pSTAT3 increase in motoneurons and sensory neurons; therefore differences in the timing of activation could not be associated with these cells. Rather than timing, our results suggest that the difference between the immature/mature phenotype of pSTAT3^+^ motoneurons could be related to the loss of regenerative capacity. We can also conclude that, as metamorphosis advances, motoneurons lose the early response to the JAK‐STAT pathway as seen by the pSTAT3 levels in motoneuron columns observed at 3 hpt in NR56 stage but at 6 dpt in NR66 stage.

### 3.3 JAK‐STAT pathway in leukocytes

In mammals, the JAK‐STAT pathway is also activated in microglia and macrophages after SCI (Park et al., 2014; Yamauchi et al., 2006). *In vitro* studies have shown that STAT3 activated microglia induce the expression of inflammatory genes on astrocytes and neurons (Molet et al., 2015), while an enhanced STAT3 signaling in myeloid cells induces *in vivo* neuro‐inflammation (Qin et al., 2012). Nevertheless, in our model the sustained pSTAT3 signal in NR66 stage does not co‐localize with CD45^+^ leukocytes. This dismisses a relation between inflammatory response and sustained activation in the non‐regenerative response.

### 3.4 Sustained JAK‐STAT regeneration reduces neurogenesis

Our results indicate a correlation between loss of the regenerative capacity and the sustained activation of the JAK‐STAT pathway. To test if the timing of activation controls the response upon SCI, we developed a *X. laevis* transgenic line to induce a sustained JAK‐STAT pathway activation in the R stage. The *Tg(HS:caSTAT3GR)* induces pathway activation but has low survival rates after treatment, hampering the induction of a sustained activation in our established SCI model. To avoid the low survival rates we developed spinal cord grafting experiments, which have been done before in *X. laevis* tadpoles to study the role of the WNT signaling pathway in spinal cord regeneration using a similar heat shock inducible transgenic cassette (Lin, Chen, & Slack, 2012). The spinal cord graft establishes rostrocaudal continuity with the host spinal cord; therefore this approach allows us to induce a sustained induction of the JAK‐STAT pathway in a regenerative process similar to the previously characterized spinal cord transection.

We analyzed gene expression to determine if JAK‐STAT sustained activation controls neuronal precursor cell, glial or neuron lineage in response to injury. Our results showed no clear trend to neuronal precursor cell or glial regulation, but the two neurogenic genes *neugenin2a* and *neurogenin3* were downregulated in response to sustained JAK‐STAT induction. Previously, we showed that these genes are upregulated in the R stage in response to SCI (Muñoz et al., 2015). Therefore our results suggest that transient activation of the pathway is necessary to allow neurogenic differentiation of Sox2/Sox3^+^ cells in the regenerative stage.

### 3.5 Sustained JAK‐STAT activation in the non‐regenerative response

STAT3 is a necessary factor for spinal cord recovery in mammals, as it promotes astrocyte response and glial scar formation, preventing secondary damage and worsening of the functional outcomes (Herrmann et al., 2008; Okada et al., 2006; Wanner et al., 2013). The JAK‐STAT pathway also has a sustained activation in mammals, as it is shown by pSTAT3^+^ astrocytes and oligodendrocyte precursor cells in the vicinity of the lesion core up to 4 months post‐injury (Hesp et al., 2015; Tripathi & McTigue, 2008). Our results suggest that activation of the JAK‐STAT pathway should be transient to allow regenerative neurogenesis. Taking this in account, a better understanding of the role of the JAK‐STAT pathway in the mammalian response for SCI should consider the timing of activation, because it is known that spinal cord ependymal cells in mammals are fated to glia and oligodendrocytes, whereas no neurogenesis is observed (Barnabé‐Heider et al., 2010; Meletis et al., 2008). Therefore, more studies should be done to determine if the JAK‐STAT pathway controls the fate of ependymal cells in mammals and to determine the efficient timing of activation of the pathway for spinal cord regeneration in mammals.

## 4 MATERIALS AND METHODS

### 4.1 Analysis of transcriptome‐wide database

A list of genes differentially expressed between the R and NR stages in response to SCI was obtained from Lee‐Liu et al. (2014). Genes were associated with a different signaling pathway with the option “Reconstruct pathway” from the KEGG database (Kanehisa, Goto, Kawashima, Okuno, & Hattori, 2004). Genes associated with the JAK‐STAT pathway were hierarchically clustered according to their log_2_(fold change) value and represented graphically using Cluster3.0 and Java Tree View (de Hoon, Imoto, Nolan, & Miyano, 2004; Saldanha, 2004).

### 4.2 Growth and manipulation of *Xenopus laevis*

Frogs obtained from Nasco (Fort Atkinson, WI, USA) were subjected to *in vitro* fertilization and raised as described previously (Gaete et al., 2012) until they achieved Nieuwkoop and Faber stage 50 (R stage), 56 or 66 (NR56 and NR66 stages). For spinal cord manipulations, tadpoles and froglets were anesthetized in 0.1% MS222 and placed on an inverted Petri dish covered by gauze. Spinal cord transection and postoperative manipulation were executed as previously described (Lee‐Liu et al., 2014; Muñoz et al., 2015, Edwards‐Faret, Muñoz, Mendez‐Olivos, Lee‐Liu, Tapia, & Larrain, 2016). Briefly, dorsal tissue was incised at the midpoint of the thoracic segment, and then the spinal cord was transversally sectioned (transection) at the midpoint between forelimbs and hindlimbs (Fig. S1A−C). In the post‐metamorphic NR66 stage, the spinal cord was accessed through the intervertebral discs of the ossified vertebrae to perform the transection. For control in all procedures, a sham operation was performed cutting only skin and muscle dorsal tissue (Edwards‐Faret et al., 2016). All animal procedures were approved by the Committee on Bioethics and Biosafety from the Faculty of Biological Sciences, Pontificia Universidad Catolica de Chile.

### 4.3 Spinal cord isolation

To isolate spinal cords, transected or sham‐operated animals at the R and NR stages were sacrificed at 3 or 6 hr post‐transection (hpt) and 1, 2, 6, 15, or 30 days post‐transection (dpt). We isolated samples as described previously (Lee‐Liu et al., 2014; Fig. S1A−C) for the R and NR66 stages. For the NR56 stage, the isolated spinal cord segment went from the transection site to the cloaca and was 3.8 mm long (Fig. S1B).

For RT‐qPCR replicates we isolated spinal cords from pools of 12−15 tadpoles at the R stage, 8−10 tadpoles at the NR56 stage and three froglets at the NR66 stage, while western blot samples included 8−10 tadpoles at the R stage, four to five tadpoles at the NR56 stage and three froglets at the NR66 stage.

### 4.4 RT‐qPCR

Total RNA was obtained as described previously (Lee‐Liu et al., 2014), to give a total of at least three biological replicates. cDNA was synthesized using M‐MLV reverse transcriptase (Promega, Madison, WI, USA), and qPCR was performed using Maxima SYBR Green (Thermo Scientific, Waltham, MA, USA). The relative expression ratio (Pfaffl, 2001) was then calculated using *eef1a1* (GenBank BC043843) as a reference gene and comparing to sham operated levels. The primers used in this study are indicated in Table S1.

### 4.5 Western blot

Spinal cords were homogenized in RIPA lysis buffer with protease inhibitors (benzamidine 1 μM; leupeptin 5 μg/ml; Na_3_VO_4_ 200 μM; sodium pyrophosphate 200 μM and phenylmethylsulfonyl fluoride 200 μM). Western blot was performed as described previously (Gaete et al., 2012; Muñoz et al., 2015); proteins were quantified with the bicinchoninic acid protein assay kit (Thermo Scientific) and 10 μg of protein was loaded in each lane. The primary antibodies used were rabbit monoclonal antibody (mAb) anti‐pSTAT3 (1:2000; Cell Signaling Technology, Danvers, MA, USA; D3A7) and rabbit polyclonal anti‐STAT3 (1:1000; Santa Cruz Biotechnology, Dallas, TX, USA; sc‐482). Densitometry analysis of pSTAT3 and STAT3β bands was performed with ImageJ (National Institutes of Health, Bethesda, MD, USA) and in each experiment the pSTAT3/STAT3 ratio was normalized to the sham or uncut control.

### 4.6 Immunofluorescence

Tissue processing and immunofluorescence was performed as described by Muñoz et al. (2015) with minor modifications. Briefly, spinal cord cryosections were permeabilized with 100% methanol at −20°C for 10 min and then with phosphate‐buffered saline (PBS) + 0.2% Triton X‐100 (PBST) at room temperature for 10 min, blocked in PBST + 10% goat serum, incubated with primary antibodies overnight at 4°C, secondary antibodies for 2 hr at room temperature and stained with TOTO‐3 or Hoechst 33342. Antibodies used were rabbit mAb anti‐pSTAT3 (1:200, Cell Signaling Technology, D3A7), mouse mAb anti‐Sox2 (1:200, Cell Signaling Technology, L1D6A2), mouse mAb anti‐Islet1/2 (1:50; Developmental Studies Hybridoma Bank, Iowa City, IA, USA; 39.4D5), mouse mAb anti‐CD45 (1:50; Xenopus laevis Resource for Immunobiology, Rochester, NY, USA; CL21) and AlexaFluor 488 or 555 (1:500; Invitrogen, Carlsbad, CA, USA) as secondary antibodies.

Samples were photographed using an inverted fluorescence microscope (Microfluo Olympus IX71) or a confocal microscope (FV‐1000 Olympus Confocal Laser Scanning Microscope). Total cell counts for quantification analysis were determined using the ImageJ cell counter plugin.

### 4.7 Transgene constructs and transgenesis

We generated a construct using the HGEM vector (Beck, Christen, & Slack, 2003), which contains a *Xenopus* HSP70 promoter to induce expression of a gene of interest and the *Xenopus* γ‐crystallin promoter driving expression of green fluorescent protein (GFP) as a visible marker for transgene insertion. The transgene sequence was obtained from a pCS2‐caSTAT3GR (Nichane, Ren, & Bellefroid, 2010). The caSTAT3GR‐polyA fragment was digested from this construct with *Hin*dIII and *Bss*HII and then cloned into the HGEM vector.

The HGEM‐caSTAT3GR construct was used to generate the transgenic line *Xla.Tg(cryga:GFP,hsp70:CAstat3‐GR)^Larra^*, named following the working guidelines for naming *Xenopus* transgenics and simplified as *Tg(HS:caSTAT3GR)*. Transgenesis was adapted from the restriction enzyme mediated integration method (Ishibashi, Kroll, & Amaya, 2009). Transgenic embryos were selected by GFP^+^ eyes and raised until sexual maturity.

An F0 male frog 7 months old was used to generate F1 tadpoles through natural mating. Once tadpoles reached the R stage, they were heat shocked (30 min at 34°C) once and incubated in 0.1× Barth with 10 μM dexamethasone to characterize survival rate and JAK‐STAT activation in the central nervous system (brain and spinal cord) by RT‐qPCR and western blot. Lastly, F2 tadpoles were generated for transplantation experiments.

### 4.8 Spinal cord transplantation

For spinal cord transplantation (Fig. S1D), the dorsal tissue of a wild‐type (WT) tadpole was longitudinally incised to expose the spinal cord. The incision was made from the midpoint between forelimbs and hindlimbs to the hindlimbs level. The exposed spinal cord was cross‐sectioned using iridectomy scissors at both ends and then removed. The 1.5–2.0 μm donor spinal cord piece was inserted into a WT tadpole, and then tadpoles were kept in 01× Barth supplemented with antibiotics and dexamethasone 10 μM, and fed every 2 days. The first heat shock was induced in the donor tadpoles 30 min before spinal cord transplantation; two other heat shocks were induced at day 2 and 4 in the host tadpoles. At day 5 the host tadpoles were sacrificed and the transplanted spinal cord was isolated again for RT‐qPCR analysis. Five replicates for RT‐qPCR were prepared from pools of 8−12 control and transgenic transplanted tadpoles.

### 4.9 Statistical analysis

Results for RT‐qPCR were analyzed by one simple *t*‐test with 95% confidence to determine a significant difference with sham levels (value 0). Results for RT‐qPCR, western blot and cell counting were analyzed by one‐way ANOVA and the Bonferroni test with multiple comparisons to compare between time points. For one‐way ANOVA test differences were considered statistically significant at *P < 0.05, **P < 0.01, ***P < 0.001, and ****P < 0.0001 for at least three independent experiments. Error bars in all figures indicate the standard error of the mean.

## 5 Conclusion

We demonstrated that spatio‐temporal changes in activation of the JAK‐STAT pathway are associated with loss of the regenerative capacity in *X. laevis*, leading to a more comprehensive characterization of the molecular and cellular differences between R and NR stage SCI. Although further studies should clarify the role of early activation on Sox2/3^+^ cells in the regenerative response, our results suggest that transient timing of activation is necessary to allow regenerative neurogenesis. Altogether these results provide novel insights on the role of the JAK‐STAT pathway function in SCI, which is known to be necessary for spinal cord recovery in mammals but where no study has assessed an efficient temporal activation of the pathway for spinal cord recovery.

## Supporting information

Figure S1. Anatomical positions for spinal cord transection and grafting approaches.
Figure S2. Auto‐fluorescence clots in NR66 stage spinal cord.
Figure S3. STAT3 activation in motoneurons and sensory neurons.
Figure S4. STAT3 activation in leukocytes.
Figure S5. Characterization of *Tg(HS:caSTAT3GR)* transgenic tadpoles.

Table S1. Primers used in this study.
