# Supplementary material for: JAK‐STAT pathway activation in response to spinal cord injury in regenerative and non‐regenerative stages of Xenopus laevis
Source: Regeneration (Oxf). 2017 Mar 14;4(1):21–35. doi: 10.1002/reg2.74 (PMC5350081; doi:10.1002/reg2.74)
Supplement: Supplementary file 1 — Figure S1. Anatomical positions for spinal cord transection and grafting approaches. Figure S2. Auto‐fluorescence clots in NR66 stage spinal cord. Figure S3. STAT3 activation in motoneurons and sensory neurons. Figure S4. STAT3 activation in leukocytes. Figure S5. Characterization of Tg(HS:caSTAT3GR) transgenic tadpoles. [file REG2-4-21-s001.pdf]

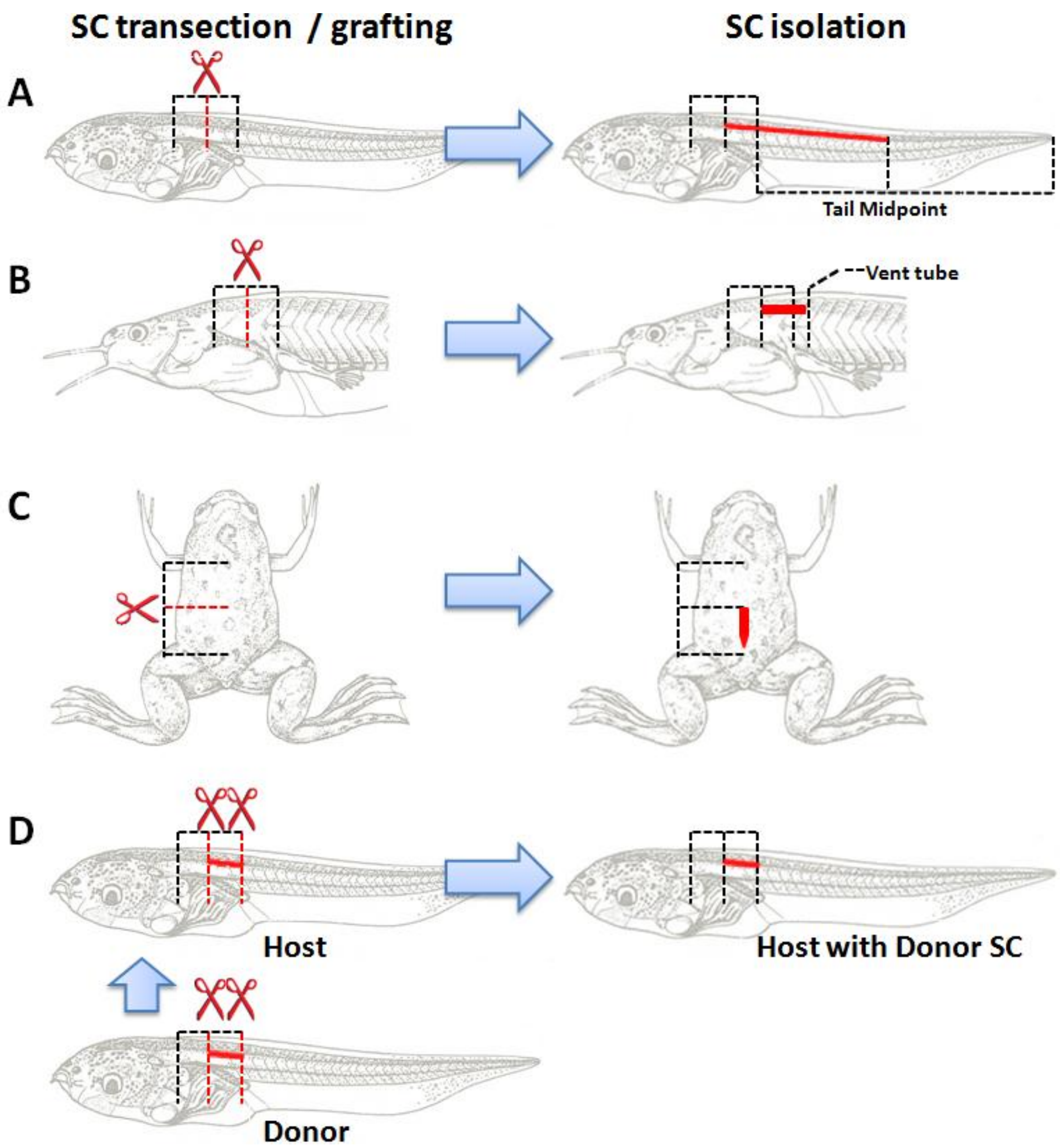

Figure S1. Anatomical positions for spinal cord transection and grafting approaches. A-C, Spinal cord (SC) transection (red scissor and dashed line) is performed at the mid-point between hindlimbs and forelimbs (black dashed lines), as indicated for (**A**) R-, (**B**) NR56- and (**C**) NR66-stages. In control groups (sham operation) an incision in skin and muscle is performed at the same level, without injuring the SC. Caudal portion of the SC is isolated from the transection site to (**A**) the mid-point of the tail in R-stage, (**B**) the beginning of the cloaca (Vent tube) in NR56-stage and (**C**) the end of the spinal cord in NR66-stage animals. **D**, SC grafting is performed by transections (red scissors and dashed lines) of donor and host in the mid-point between limb buds and in the forelimb buds. SC isolation is performed at the same sites.

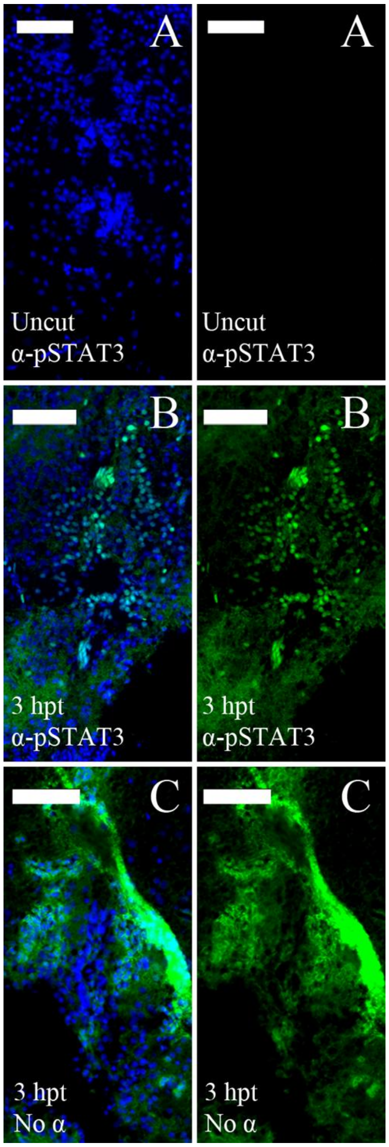

Figure S2. Autofluorescence clots in NR66-stage spinal cord. Coronal sections through the spinal cord of NR66 animals with focus in the ventricular zone, analyzed for pSTAT3 (green) and Hoescht (nuclei, blue). **A-A'**, section from an uninjured animal. **B-C'**, sections from an animal in the spinal cord stump at 3 hpt. **B-B'** shows nuclear signaling of pSTAT3 and **C-C'** is the control without primary antibody. White bars indicate 50 µm.

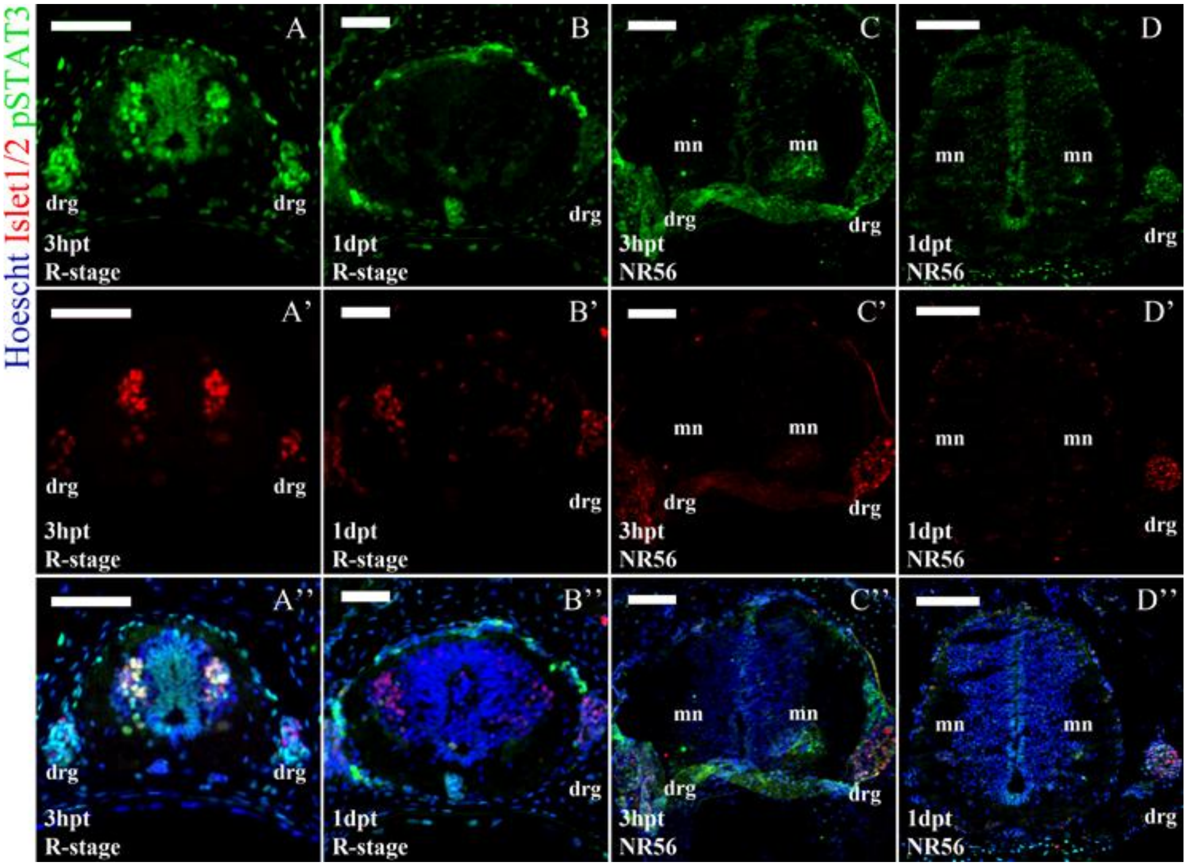

Figure S3. STAT3 activation in motoneurons and sensory neurons. Coronal sections through the spinal cord of R- and NR56-stage animals, analyzed for pSTAT3 (green), Islet1/2 (red) and Hoescht (nuclei, blue). Motoneuron columns are indicated by mn and dorsal root ganglia by drg. **A-B''**, sections from R-stage animals at 3hpt (**A-A''**) and 1dpt (**B-B''**). **C-F''**, sections from NR56-stage animals at 3hpt (**C-C''**) and 1dpt (**D-D''**). White bars indicate 50 µm (**A-B''**) and 100 µm (**C-D''**).

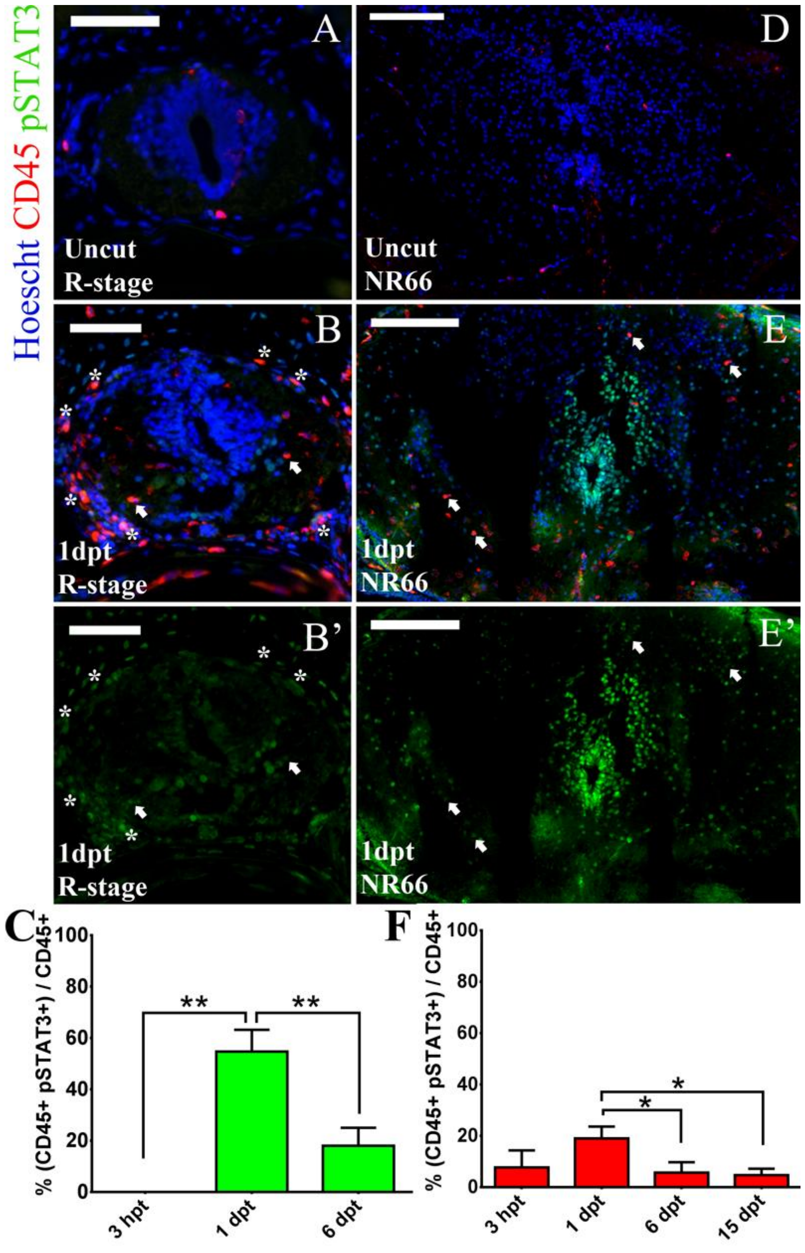

Figure S4. STAT3 activation in leukocytes. Coronal sections through the spinal cord of R- and NR66-stage animals, analyzed for pSTAT3 (green), CD45 (red) and Hoescht (nuclei, blue). Leukocytes pSTAT3+ are indicated by white asterisks in the meninges and by white arrows in the spinal cord. **A-B′**, sections from an uninjured R animal (**A**) and at 1dpt (**B-B′**). **C-E’**, sections from an uninjured NR66 animal (**C**) and at 1dpt (**E-E’**). White bars indicate 50 μm in A-B', 100 μm in C-D and 30 μm in E-F. **C-F**, quantification of pSTAT3$^+$ CD45$^+$ cells in three animals per time point was performed for (**C**) R- and (**F**) NR66-stages. Difference between time points was assessed by multiple comparisons with a one-way ANOVA test (P value **>0,01 and *<0,1).

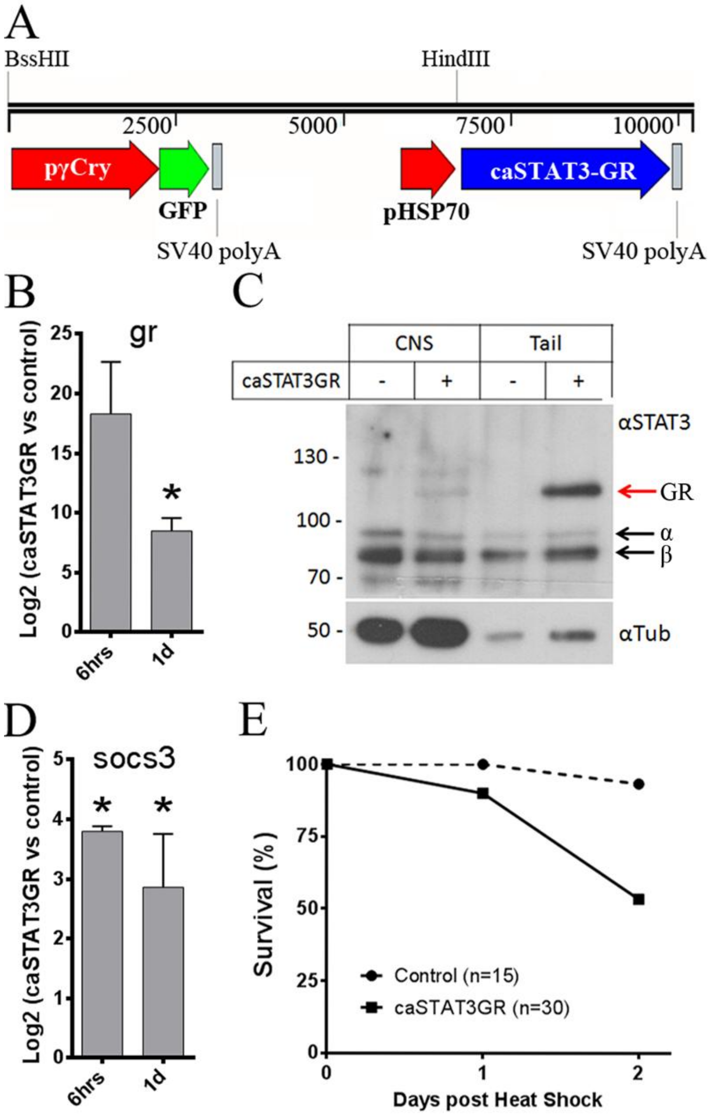

Figure S5. Characterization of *Tg(HS:caSTAT3GR)* transgenic tadpoles. A. Transgenic construct used in this study. caSTAT3GR was inserted in an HGEM construct by HindIII and BssHII restriction enzymes. **B-E**, F1 transgenic animals and their control siblings were raised to stage 50, heat shocked once and incubated with dexamethasone during 2 days. **B**, RT-qPCR analysis shows *gr* up-regulation in the central nervous system (CNS) 6 hours and 1 day post-treatment **C**, Western blot analysis shows presence of caSTAT3GR protein (red arrow, GR) in comparison to total STAT3 (black arrows, α and β isoforms) at 8 hours post-treatment in the CNS and in the tail. **D**, RT-qPCR analyses shows *socs3* up-regulation in the CNS after treatment. **E**, Tadpoles expressing caSTAT3GR had a reduced survival rate in comparison with their controls siblings. For RT-qPCR analyses, one sample t-test with 95% confidence was performed to determine a significant difference (* symbol) from control tadpoles, using at least 3 independent replicates.
