# Supplementary material for: JAK‐STAT pathway activation in response to spinal cord injury in regenerative and non‐regenerative stages of Xenopus laevis
Source: Regeneration (Oxf). 2017 Mar 14;4(1):21–35. doi: 10.1002/reg2.74 (PMC5350081; doi:10.1002/reg2.74)
Supplement: Supplementary file 2 — Table S1. Primers used in this study. [file REG2-4-21-s002.pdf]

Table S1. Primers used in this study.

Gen	Forward	Reverse
eef1a1	ACGCGTGGGTAAGTGTCCACC	GGCCAGTTGTTGTGGACTTTCCAG
socs3	TGCCTGACCCAGGGATCTTA	AAACACACAATTCCCGCAGC
leptin	GTGACATGGAGAACCTCCGC	GGCTGTGTTTTCTGGCTGTG
lif	TTTGTGTGCACTTTGGAGCG	TGCAGAGCACTTGTGCCTAA
gr	GTTCCTGCAACGTTACCACA	TCTGGAACAGAGCTATCATATCC
